# Quality Evaluation of Chicken Liver Pâté Affected by Algal Hydrocolloids Addition: A Textural and Rheological Approach

**DOI:** 10.3390/ani14182715

**Published:** 2024-09-19

**Authors:** Ladislav Šiška, Robert Gál, František Štefunko, Zdeněk Polášek, Zuzana Lazárková, Markéta Pětová, Zdeněk Trvdoň, Richardos Nikolaos Salek

**Affiliations:** 1 Department of Food Technology, Faculty of Technology, Tomas Bata University in Zlin, nam. T.G. Masaryka 5555, 760 01 Zlin, Czech Republic; siska@utb.cz (L.Š.); f_stefunko@utb.cz (F.Š.); zpolasek@utb.cz (Z.P.); lazarkova@utb.cz (Z.L.); rsalek@utb.cz (R.N.S.); 2 Laboratory of Food Quality and Safety Research, Department of Logistics, Faculty of Military Leadership, University of Defence, Kounicova 65, 662 10 Brno, Czech Republic; marketa.petova@unob.cz; 3 Schrom Farms spol. s.r.o., 742 91 Velké Albrechtíce, Czech Republic; zdenek.tvrdon@schomfarms.cz

**Keywords:** chicken liver pâté, carrageenan, furcellaran, viscoelastic properties, textural properties

## Abstract

**Simple Summary:**

Chicken liver pate is a smooth and spreadable product made from chicken meat, chicken livers, fat, seasoning and other optional ingredients (such as hydrocolloids). Hydrocolloids (polysaccharides or proteins) are commonly used in spreadable meat/poultry products (such as chicken liver pâté) to improve texture, stability and moisture retention, resulting in a product with desired functional and organoleptic properties. In general, the chicken liver pâté samples showed their highest hardness and viscoelastic moduli values when κ-carrageenan and furcellaran were used. Suggestions for producing a softer chicken liver pâté consistency involve utilizing ι-carrageenan at a level between 0.25% and 0.75% (*w*/*w*) or furcellaran at a 0.25% *w*/*w* concentration. We can conclude that the use of hydrocolloids in the manufacturing of chicken liver pâté could be an effective solution leading to the development of products with desired techno-functional properties.

**Abstract:**

Hydrocolloids are used in spreadable meat or poultry products to improve consistency, emulsion stability and water retention, resulting in products with desired functional and organoleptic properties. The scope of the work was to evaluate the addition of three divergent algal hydrocolloids (κ-carrageenan, ι-carrageenan, furcellaran) at four different concentrations (0.25, 0.50, 0.75, and 1.00% *w*/*w*) on the physicochemical, textural, rheological and organoleptic properties of model chicken liver pâté (CLP) samples. Overall, the highest hardness and viscoelastic moduli values of the CLP samples were reported when κ-carrageenan and furcellaran were utilized at a concentration of 0.75% *w*/*w* (*p* < 0.05). Furthermore, increasing the concentrations of the utilized hydrocolloids led to increase in the viscoelastic moduli and hardness values of CLP. Compared to the control sample, an increase in spreadability was reported in the CLP samples with the addition of hydrocolloids. Finally, the use of algal hydrocolloids proved to be an effective way to modify the techno-functional properties of CLP.

## 1. Introduction

Spreadable poultry products such as chicken liver pâté (CLP) have a significant gastronomic tradition and are generally considered valuable in terms of nutritional and organoleptic characteristics. Generally, the basic ingredients of pâtés are ground meat (the grain size typical of the type of product) and fat, commonly mixed with divergent minor additives (spices and other optional ingredients) which are considered necessary to achieve the desired functional and organoleptic properties of the final product. The above-mentioned spreadable product has a texture that can vary from smooth and creamy to dense and firm or coarse and chunky. Furthermore, this product group can also be defined as an emulsified meat/poultry product [1,2,3,4].

However, with the worldwide increase in chicken production and consumption, the poultry industry produces various by-products, such as chicken liver and skins, which are generally under-utilized. To deal with this issue, new strategies should be tested. In particular, the use of the above-mentioned by-products can lead to their transformation into usable and processable food raw materials [5]. This trend is desirable from several points of view, both as a contribution to consumers and to strategies for greater process efficiency and sustainable development linked to mitigating the negative environmental impacts of industrial activity [6,7]. One of the general problems that can occur in novel food products of animal origin using by-products as raw materials is the change or deterioration of the product’s functional properties (textural, rheological, organoleptic) [2,8]. Thus, one of the possible options to enhance the functional and organoleptic properties of such novel food products is the application of different types of hydrocolloids [9].

In general, the term hydrocolloid is commonly used to refer to a range of biopolymers (polysaccharides and proteins) that are widely used in various food sectors to perform as emulsifying, gelling, and stabilizing agents. In particular, the food industry has seen a large increase in using these substances in recent years. Although hydrocolloids are often present only in low concentrations, they can have a significant effect on the textural and organoleptic properties of food products [9,10,11]. Furthermore, an important property of hydrocolloids, of great interest to the meat industry, is their ability to bind water and form a gel. In addition, hydrocolloids are used as another gelling system leading to improved yield and rheological properties and thus reducing production cost [8,9,12,13].

Carrageenans are one of the most frequently used hydrocolloids in studies and applications in this respect. Carrageenan is the generic name for a group of linearly sulfated polysaccharides extracted from red seaweeds (*Rhodophycae*; species *Kappaphycus*, *Eucheuma*, *Chondrus*, *Gigartina* a *Chondracanthu*) and consisting of alternating residues of 1,3-linked β-d-galactose (G-units) and 1,4-linked α-d-galactose (D-units), which may be partially or completely in the form of 3,6-anhydro-derivative (DA-units) [12,13,14]. Algae commonly used in the commercial production of carrageenan include *Euchema cottonii* and *Euchema spinosum*. *Euchema cottonii* is a source of κ-carrageenan, and *Euchema spinosum* is a source of ι-carrageenan. *Furcellaria* and *Chondrus crispus* species are used as a source of κ-, and λ-carrageenan, and furcellaran [10]. Three classes of carrageenans are important in the food industry, and according to their chemical composition, they are divided into the following fractions: κ-, ι-, and λ-carrageenan. Their differences are due to the presence/absence of 3,6-anhydrogalactose in the 1,4-linked residue and the number and location of sulfate groups [12,13,14,15]. Kappa-carrageenan is soluble in hot water (˃65 °C) and in the presence of calcium ions can form strong and brittle gels. In the presence of potassium ions, it forms more elastic gels. Iota-carrageenan in its sodium salt form is slightly more soluble in cold water than κ-carrageenan and in the presence of calcium ions forms a soft gel [15,16,17]. When describing carrageenans’ importance for the food industry, furcellaran should also be mentioned. Furcellaran is typically obtained from the extract of red algae (*Furcellaria lumbricalis*), which resembles both agar and κ-carrageenan in its properties [15]. Furcellaran is a sulfated, negatively charged polysaccharide (galactan) and its fragment consists of (1→3) β-d-galactopyranose with a sulfate group at C-4 and (1→4) 3,6-anhydro-α-d-galactopyranose. Furcellaran is theoretically defined as one ester sulfate group per tetramer at the fourth position of the galactose unit. Structurally, furcellaran is related to κ-carrageenan, with the main basic difference being that furcellaran is less sulfated [12,18,19,20,21,22].

However, there is a lack of information on the influence of different algal hydrocolloid additions on the consistency of CLP, which is typically assessed using rheological and textural parameters. Similarly, we are unaware of any study comparing the impact of furcellaran application on the characteristic properties of CLP to the application of other carrageenan fractions (kappa, iota). Based on the above, the current study aimed to formulate CLP using chicken by-products (chicken liver and skins) and three different algal hydrocolloids (κ-carrageenan, ι-carrageenan and furcellaran) in different concentrations (0.25, 0.50, 0.75 and 1.00% *w*/*w*) and evaluate the physicochemical, textural, and rheological properties of CLP.

## 2. Materials and Methods

### 2.1. Raw Materials for the Manufacture of Chicken Liver Pâté Samples

Chicken thigh fillet (muscle without skin), chicken liver and chicken skin were used to prepare the CLP samples. These raw materials were obtained from the local market (LUKROM s.r.o., Lípa, Czech Republic). These chicken slaughter by-products were from *Gallus domesticus*, Ross 308, female, age 35 days, slaughter weight approx. 1.3–1.5 kg. The birds were housed in floor pens and reared in controlled environmental conditions (fully compliant with Ross 308 chicken standards). The diets used contained the recommended nutrient composition according to the Ross 308 Chicken Breeding Guidelines [23]. The birds were slaughtered at a local slaughterhouse (Electrical water bath stunning was used before bird slaughtering; RACIOLA Uherský Brod, s.r.o., Uherský Brod, Czech Republic) with a cutting plant (Veterinary Approval for EU N° CZ 8022). Nitrite curing salt (Praganda^®^, K + S Czech Republic a.s., Prague, Czech Republic), seasoning mixture (RAPS GmbH & Co. KG, Kulmbach, Germany), κ- carrageenan, ι- carrageenan (Sigma-Aldrich^®^, Merck KGaA, Darmstadt, Germany) and furcellaran EstGel1000™ (Est-agar a.s., Kärla village, Estonia) were also applied for the manufacture of the model CLP samples. The raw material composition of the CLP model samples is presented in Table 1.

### 2.2. Manufacture of the Chicken Liver Pâté Samples

The model CLP sample manufacture process (Supplementary Figure S1) started by stiffening chicken skins and boiling them in water for 30 ± 2 min. After stiffening, the skins were removed from the broth and allowed to cool at room temperature for 10 min. The broth was left at room temperature for 10 min to cool slowly. The fat fraction that floated to the surface of the broth during cooling was mechanically removed from the broth with a perforated ladle. This was followed by manufacture, which included the simultaneous production of two mixtures, namely mixture A and mixture B. Both mixtures were produced simultaneously on two identical Thermomix Vorwerk^®^ TM31 (VORWERK CS k.s., Prague, Czech Republic) blender cookers. Chicken liver with curing salt (mixture A) was mixed for 2 min using the blender cooker device at 3100 rpm. The chicken livers were pre-cut into cubes (with an approximate edge length of 1 cm) before homogenization. The stiffened chicken skins were also pre-cut into strips (approximately 1 cm wide) and divided into two equal halves. Simultaneously, on a second blender cooker device of the same type, the chicken thigh muscle was pre-cut into cubes (with an approximate edge length of 1 cm). Then, the first half of the pre-cut stiffened skins and the seasoning mixture were mixed for 30 s. Subsequently, the second half of the proportion of stiffened and cut chicken skin was added to the resulting mixture. The duration of mixing was again 30 s. Finally, broth from the skins was added to the mixture, which was again mixed for a period of 30 s. Mixture B was produced in this way. Mixture A and the proportion of hydrocolloid were added to the prepared mixture B. The final mixture was obtained using the Vorwerk^®^ TM31 device (control level 6, for 3 min). The obtained final mixture was divided into glass containers (Vetropack Moravia Glass a.s., Kyjov, Czech Republic; glass height 6.5 cm, glass volume 0.3 L), which were filled to approximately ¾ of the glass container volume. For each type of CLP, 8 glass containers were filled. The net weight of the contents inside the glass container was 150 ± 5 g. Before sealing with caps (TO 82; Tecnocap s.r.o.; Střížovice; Czech Republic), the glass containers with the non-thermally treated sample were placed in a vacuum packer (Henkelman, Mini jumbo, The Netherlands) to remove any air cavities. The edges of the containers were then cleaned, capped and properly labeled for identification purposes. Marked, capped glass containers were placed in a Rational convection oven (SelfCookingCenter^®^, SCC WE 61; RATIONAL Czech Republic s.r.o., Prague, Czech Republic; operating at 99 °C and 90–100% relative humidity) for heat treatment. The target temperature in the product’s core inside the glass container was 70–72 °C for 10 min. Temperature was controlled using a probe placed directly into the CLP sample. The cooling of the model CLP samples was realized in an ice bath until a temperature of 4 ± 1 °C in the center of sample was reached (approx. for 60 min). The cooled products were stored at 6 ± 1 °C before subsequent analyses were performed. A control sample (CS) without any hydrocolloid addition was also manufactured. A total of 39 CLP model samples (*n* = 39) were manufactured after conducting the experiment three times.

### 2.3. Physicochemical Analysis

The determinations of total protein, dry matter, total lipid contents and pH values were performed according to procedures following the ISO 937:2023 [24], ISO 5534:2004 [25], ISO 1443:1973 [26] and ISO 11289:1993 [27]. The analyses were performed six times (*n* = 6).

The determination of water activity value was performed according to ISO 18787:2017 [28]. The determination of emulsion stability (or water binding capacity) was determined according to Foegeding and Ramsey [29]. Emulsion stability (ES; rel. %) was determined from the following Equation (1):

(1) $$ES=\frac{m_{2}}{m_{1}}\times 100$$

where m_1_ (g) is the mass of the CLP model sample placed in the tube and m_2_ (g) is the mass of the sediment after draining excess liquid. The analyses were performed six times (*n* = 6).

### 2.4. Texture Profile Analysis and Spreadability Test

The textural properties of food are among the important characteristics of food quality [1,2]. The textural properties of the CLP samples were measured on a TA.XTplus analyzer (Stable Micro Systems Ltd., Godalming, UK; software used: Exponent Lite software version 4.0.13.0). The samples (cylindrical shape; with a diameter of 35 mm and a height of 10 mm) were compressed (during two cycles) to 50% of their original height using a cylindrical probe (diameter of 100 mm), with a probe speed of 1 mm/s and a trigger force of 5 g (at 20 °C ± 2 °C). The textural attributes of hardness, relative adhesiveness, cohesiveness, elasticity and gumminess were evaluated. Definitions of the above-mentioned textural attributes are reported in ISO 11036:2020 and ISO 5492:2008 [23,24,25,26,27].

Spreadability was determined according to Vincová et al. [28] with a cone-shaped main probe (male; 90°) and Plexiglas cone-shaped analyte holders (female) using a TA.XTplus analyzer (Stable Micro Systems Ltd., Godalming, UK; software used: Exponent Lite software version 4.0.13.0). CLP samples were placed in the lower cone (female), and the excess sample was removed using a spatula. The tested CLP samples were subsequently penetrated by the upper cone at a 45° angle (penetration rate 1.0 mm/s at a depth of 2.0 mm). During the measuring process, the sample tended to flow out at a 45° angle, and the spreadability was determined by the ease of its flow. In both texture profile analysis and spreadability tests, for each attribute examined, a minimum of 6 samples of CLP were used on average for the statistical analysis (*n* = 6).

### 2.5. Rheological Analysis

Rheological analysis was performed using a dynamic oscillatory shear rheometer (Kinexus; Netzsch-Gerätebau GmbH, Selb, Germany). The analysis was performed at 20.0 ± 0.1 °C in the linear viscoelastic region using a parallel plate-plate geometry (diameter 35 mm, gap 1 mm) and a shear stress amplitude of 20 Pa. During analyses, the exposed edge of the geometry was covered with a thin layer of silicone oil to prevent dehydration of the CLP samples. Elastic (G′; Pa) and viscous (G″; Pa) moduli were determined in the frequency range of 0.01–10.00 Hz. Analyses of each sample were repeated three times (*n* = 3). From the values of G′ and G″ the value of tan δ (at a frequency of 1 Hz) was calculated according to the following Equation (2).

(2) tanδ=(G″/G′)

A temperature sweep test was used to monitor the samples’ viscoelastic properties’ development during the heating and cooling stages, respectively. During the measurements, the following temperature profile was applied: (i) increase in temperature from 4.5 ± 0.25 °C to a temperature of 70 ± 0.6 °C (linear heating rate of 33 min, i.e., ≈ 2.1 °C/min), (ii) holding at the temperature 70 ± 0.6 °C (11 min), (iii) cooling to a temperature of 5 ± 0.25 °C (linear heating rate of 34 min, i.e., ≈ −2.2 °C/min). In particular, during the test, the temperature changed over time at a constant frequency of 1 Hz. The elastic (G′; Pa) and viscous (G″; Pa) moduli were recorded and tan δ (dimensionless) was calculated according to Equation (2). Each CLP sample was measured at least three times (*n* = 3).

### 2.6. Instrumental Color Analysis

The color properties of the CLP samples were analyzed using an UltraScan^®^ Pro spectrophotometer (Hunter Associates Laboratory, Inc., Reston, VA, USA). The CIE L*a*b color space with illuminant D65 (standard daylight) and an angle of 10° was used. The parameters L* (luminosity; (0—black, 100—white), a* (chromaticity on the green to red axis; from green − a* to red + a*), and b* (chromaticity on the blue to yellow axis; from blue − b* to yellow + b*) were determined according to the International Commission on Illumination. Spectrophotometer calibration was performed in the reflectance mode using white (C6299) and black (C6299G) reference tiles. Each CLP sample was measured at least six times (*n* = 6). Instrumental color analysis methods for meat and meat products were previously described by Tapp et al. [30].

### 2.7. Statistical Analysis

The use of parametric tests was denied because the normal distribution (Shapiro–Wilk test; significance level of 0.05; Minitab^®^ 16 software; Minitab Ltd.; Coventry, UK) was not acceptable for all results (*p* < 0.05). Hence, the results obtained were processed using the non-parametric analysis of variance of the Kruskal–Wallis and Wilcoxon tests (Minitab^®^ 16 software; Minitab Ltd.; Coventry, UK), with the significance level set at 0.05. The effects of hydrocolloid type and concentration were evaluated separately. In addition, 2-way ANOVA was used for evaluation of the interaction effect of the independent variables (hydrocolloid type and hydrocolloid level) on the observed quality parameters of the CLP.

## 3. Results and Discussion

### 3.1. Physicochemical Analysis

The results of the physicochemical analyses of the CLP samples are shown in Table 2. In general, no significant influence of the type or concentration of the hydrocolloid used on the moisture level, protein content, fat content, and pH level of the final product was found (*p* ˃ 0.05). In particular, the moisture value ranged from 69.76 ± 0.09% *w*/*w* to 70.68 ± 0.16% *w*/*w*, the protein content ranged from 14.25 ± 0.09% *w*/*w* to 15.06 ± 0.01% *w*/*w*, and the fat content ranged from 37.35 ± 0.28% *w*/*w* to 40.17 ± 0.71% *w*/*w*. Furthermore, the pH values were in the range of 6.87 ± 0.02–6.94 ± 0.02. Similar conclusions were previously reported by Delgado et al. [31], and Petcharat et al. [32]. According to Warner et al. [33] and Zhang et al. [34], the functional properties of food can be affected by the pH value. However, we did not detect a significant effect of the type of hydrocolloid used on the pH values (*p* ˃ 0.05).

The obtained water activity (a_w_) values ranged from 0.994 ± 0.002 to 0.998 ± 0.001. Mutual deviations in a_w_ between individual samples in relation to the type or concentration of the hydrocolloid used were not significant (*p* ˃ 0.05). The measured a_w_ values correspond to the typical values for meat and meat products as reported by Berk [35]. Moreover, comparing our findings with the results presented by Rodel et al. [36], a_w_ values cannot be considered a significant barrier mechanism preventing the growth of microorganisms, leading to products in which spoilage caused by possible microbiological contamination can be reasonably assumed.

Moreover, the ES values ranged from 99.32 ± 0.04% rel. to 99.97 ± 0.02% rel. (*p* ˃ 0.05). Compared to the study of Pires et al. [37], who investigated the emulsion stability of reformulated meat products (Bologna sausage), our measured ES values were higher. The stability of the emulsion can thus be assessed as relatively high. Information has been published on the usefulness of polysaccharides, including carrageenans, as functional hydrocolloids during meat processing and production of meat products, and in this context, they properly bind water [38]. Additionally, Verbeken et al. [39] stated a better ability of muscle protein to bind water in the presence of carrageenan. At the same time, however, our results do not contradict the study of Foegeding and Ramsay [29] which does not consider the effect of the use of carrageenans on the water-binding capacity to be very significant.

### 3.2. Texture Profile Analysis and Spreadability Test

**Figure 1:**
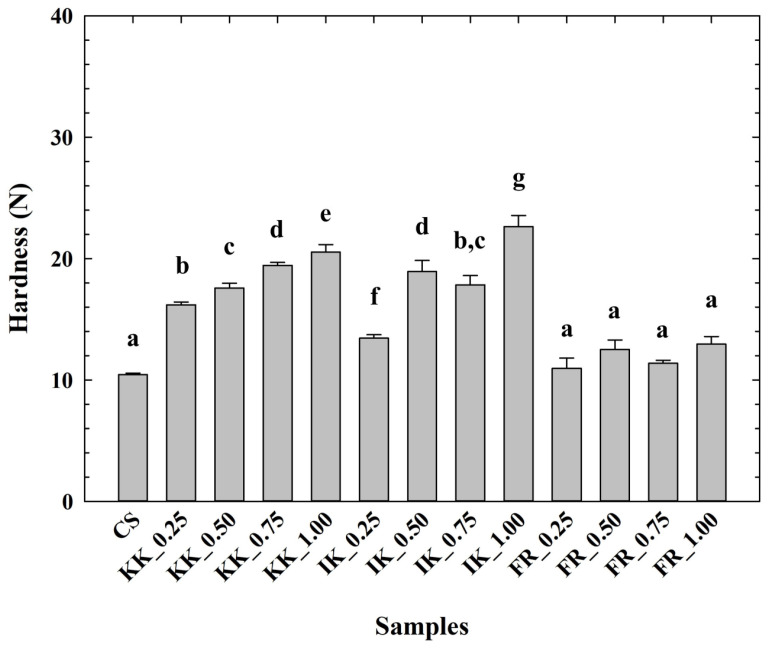
Development of chicken liver pâté hardness (calculated as the maximum force, N) depending on the type and concentration of hydrocolloid [κ-carrageenan (KK); ι-carrageenan (IK); furcellaran (FR); no hydrocolloid addition (control sample—CS); 0.25% *w*/*w*; 0.50% *w*/*w*; 0.75% *w*/*w*; 1.00% *w*/*w*; *n* = 9; the results were expressed as means (columns) and standard deviations (bars)]. Different letters (a–g) indicate significant differences at *p* < 0.05; error bars represent the standard deviation.

The hardness values of the CLP are depicted in Figure 1. From the obtained results, it can be reported that the CLP samples with hydrocolloid addition presented higher values of hardness compared to the CS (*p* < 0.05). Furthermore, there was an observable increase in the hardness value with the increasing concentrations of the applied hydrocolloids in the tested CLP samples (*p* < 0.05). The lowest hardness values were reported for the CS. On the contrary, the addition of carrageenans at 1.00% *w*/*w* significantly affected the increase in hardness (*p* < 0.05). In particular, there was a specific dependence and several differences in the modification of the textural properties of the evaluated CLP according to the type of hydrocolloid (κ-carrageenan, ι-carrageenan, and furcellaran) and, at the same time, according to the utilized concentration level (the amount) of hydrocolloid in the model samples. The effect of the application of carrageenans (iota or kappa) on the hardness development of the CLP manufactured in this study showed a more pronounced effect in relation to the effect of furcellaran.

Furthermore, in the case of the application of furcellaran, with the increasing concentration of the hydrocolloid, a trend in increasing cohesiveness and gumminess of the samples can be observed (Table 3). In the case of model samples with the addition of κ-carrageenan and samples with the addition of ι-carrageenan, this trend was less intense. For samples in which furcellaran was applied, compared to samples in which κ-, or ι-carrageenan was used, the obtained data further showed an increase in relative adhesiveness up to a concentration level of 0.75% *w*/*w*. From this point of view, the application of furcellaran at a concentration level of 0.5% *w*/*w* appears interesting when the achieved value of relative adhesiveness is comparable to the maximum value of relative adhesiveness, which was achieved with the application of 0.75% *w*/*w* fucellaran. At the application level of 1.00% *w*/*w* of furcellaran, the increase in relative adhesiveness did not continue, and a decrease followed. Additionally, the relative adhesiveness of the CLP samples with κ-carrageenan increased the most at a concentration of 1.00% (*w*/*w*) and for the sample with ι-carrageenan at 0.75% (*w*/*w*). Our findings correspond with those previously reported by Polášek et al. [9], who also concluded that increasing the content of furcellaran and κ-carrageenan increased the values of hardness and gumminess. Furthermore, according to the latter authors, the increase in these values could be attributed to the possible interactions between hydrocolloid molecules and the protein matrix [9]. These interactions can occur between the carrageenan molecules and the negatively charged carbonyl groups on the protein through a cationic bridge, or it can be a direct interaction between the carrageenan molecules and the positively charged amino groups of the present protein. Additional interactions like hydrogen bonds and hydrophobic or covalent bonds might play a role in supporting the protein-polysaccharide structure [40]. As mentioned by Ayadi et al. [41], another explanation might involve the creation of a supplementary gel network due to the presence of polysaccharides. Meat proteins can form a compact gel network in which carrageenan (κ-, or ι-) or furcellaran remain in discrete regions, probably in the interstitial spaces of that protein network. Thus, a continuous carrageenan/furcellaran gel network can be formed due to the interactions between the polysaccharide and the existing protein gel network. In addition, Ruusunen et al. [42] reported that the conformational structures can vary due to the number of sulfate groups present in the polysaccharide molecule, which can affect the hardness values of the final product. This can explain the differences in the effect of the application of κ-carrageenan, ι-carrageenan and furcellaran in the tested CLP. Petcharat et. al. [32] investigated the effect of the addition of different forms of furcellaran on the properties of sardine surimi, and an increase in hardness, gumminess and chewiness as a result of increasing the concentration of furcellaran was reported. The increase in gel strength could be explained by the electrostatic interactions of furcellaran with myofibrillar proteins [9].

**Figure 2:**
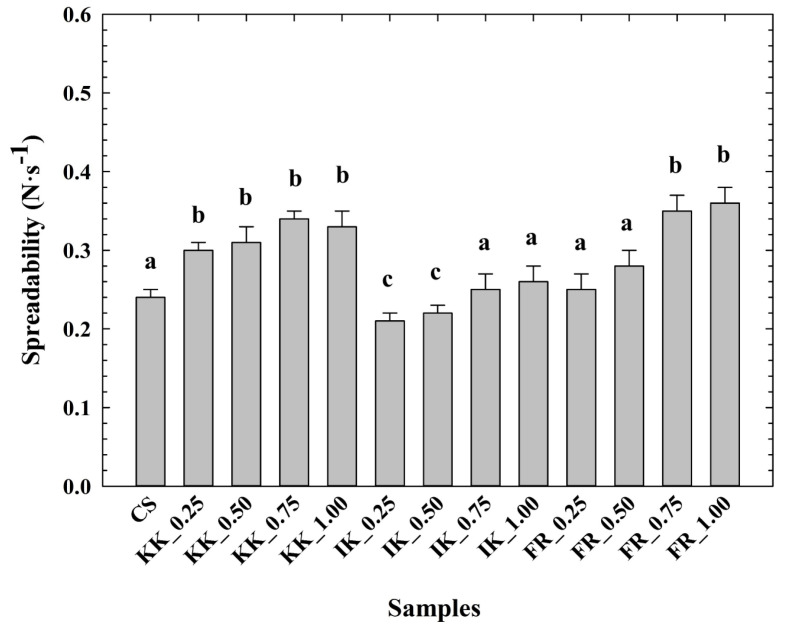
Development of chicken liver pâté spreadability (N·s^−1^) depending on the type and concentration of hydrocolloid [κ-carrageenan (KK); ι-carrageenan (IK); furcellaran (FR); no hydrocolloid addition (control sample—CS); 0.25% *w*/*w*; 0.50% *w*/*w*; 0.75% *w*/*w*; 1.00% *w*/*w*; *n* = 6; the results were expressed as means (columns) and standard deviations (bars)]. Different letters (a–c) indicate significant differences at *p* < 0.05; error bars represent the standard deviation.

The determined spreadability values of the CLP samples are shown in Figure 2. In general, the spreadability values were similar for all tested samples. Compared to the CS, an increase in spreadability was evident in the CLP samples with the addition of hydrocolloids. Moreover, the lowest spreadability values were reported for the sample with the addition of ι-carrageenan (0.25–0.50% *w*/*w*). In contrast, the spreadability values for the CLP with the use of κ-carrageenan and furcellaran were higher. However, Tonchev et al. [43], reported higher spreadability values. The latter authors used a different raw material composition.

### 3.3. Rheological Analysis

**Figure 3:**
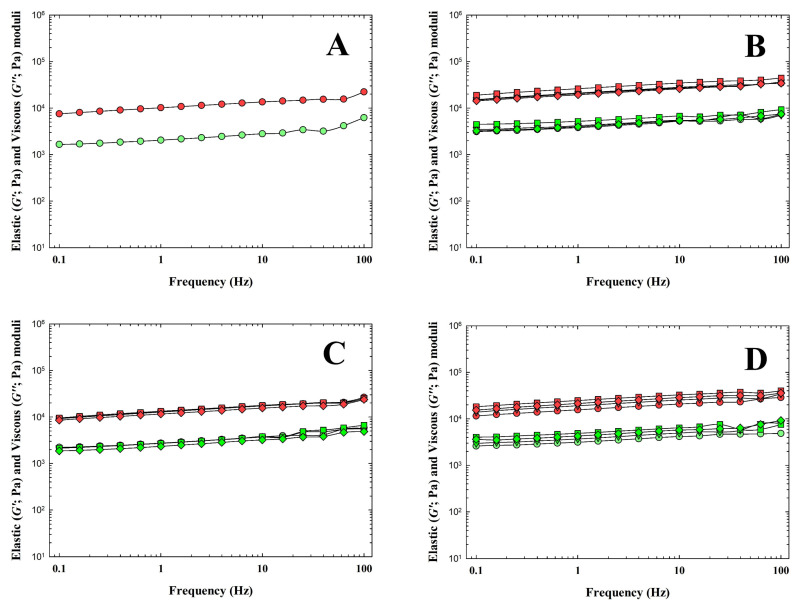
Dependence of the elastic (G′; red symbols; Pa) and the loss (G”; green symbols; Pa) moduli of chicken liver pâté samples manufactured with κ-carrageenan (**B**), ι-carrageenan (**C**) and furcellaran (**D**) in the concentrations of 0.25% (circle), 0.50% (triangle), 0.75% (square) and 1.00% (rhombus) *w*/*w* on the frequency (f; in the range of 0.1–100.0 Hz). The control sample (without any hydrocolloid addition) was also included (**A**).

In general, food viscoelastic properties can be characterized by the use of the elastic (G′) and viscous (G″) moduli. The G′ modulus determines the degree of elasticity of the material, and the G″ modulus determines the degree of viscous behavior and the ability of the material to dissipate energy [44,45]. Results of rheological analysis (frequency sweeps) of the CLP samples are shown in Figure 3. From the obtained results, it can be reported that with the increasing amount of all tested hydrocolloids, the G′ modulus increased up to a hydrocolloid concentration value of 0.75% (*w*/*w*) (*p* < 0.05). Furthermore, it is worth noting the relatively high value of the G′ modulus value of the CLP with 0.25% (*w*/*w*) κ-carrageenan (*p* < 0.05). Moreover, the addition of κ-carrageenan had the greatest effect of all the hydrocolloids on the increase in the G′ and G″ values compared to the CS (*p* < 0.05). In contrast, the lowest effect on the increase in the viscoelastic moduli values resulted from the addition of ι-carrageenan. Generally, based on the results of the current study, the CLP presented a more elastic character (G′ ˃ G″). Similar results were also reported by Delgado et al. [31] and Polášek et al. [9]. The differences in the effect of the added hydrocolloid with the type of carrageenan used, which we found, further correspond with the study by Brenner et al. [46]. The effects of individual types of hydrocolloids (κ-carrageenan, ι-carrageenan, furcellaran) of the same concentration and the amount of addition of hydrocolloid in the CLP product on the values of the viscoelastic moduli are different depending on the type of hydrocolloid. Our results also confirmed a lower effect of the addition of ι-carrageenan compared to the addition of κ-carrageenan or furcellaran at the same concentration. Moreover, Brenner et al. [46] investigated the rheological properties of mixed gels of κ-carrageenan, ι-carrageenan and stated that the effect of κ-carrageenan on the G′ values is much greater than the effect of ι-carrageenan with the addition of a similar amount of the latter. Based on the results obtained, we can further agree with the study by Feng et al. [47], who reported that the addition of hydrocolloids promoted the denaturation and aggregation of meat proteins, which played a crucial role in the subsequent formation of a three-dimensional gel network. Moreover, Lesiow et al. [48] further emphasized, in this context, that at lower temperatures, pre-formed protein aggregates play an important role in the subsequent formation of a gel during the thermal heating of the product. Cao et al. [49] reported that κ-carrageenan has no effect on the denaturation of meat proteins and acts only as a physical filler. On the contrary, Feng et al. [47] stated that κ-carrageenan is an active filler that can induce a strong interaction between fillers and proteins in the meat matrix, thereby leading to higher G′ values.

**Figure 4:**
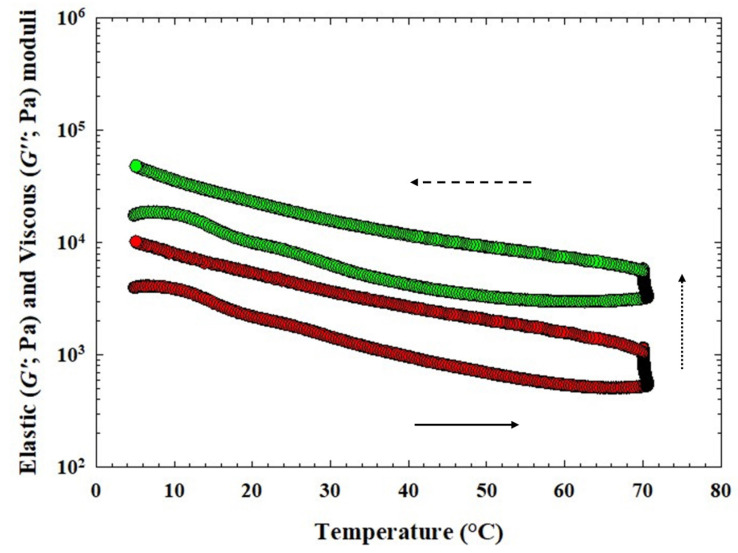
Development of the elastic (G′; green circles) and the viscous (G″; red circles) moduli of chicken liver pâté samples during heating (bottom part of the curve; the direction of the arrow (solid) presents the temperature increase), holding at 70 °C (presented with the dot arrow) and cooling [upper part of the curve; the direction of the arrow (dash) presents the temperature decrease] of the control sample (CS; without any hydrocolloid addition).

**Figure 5:**
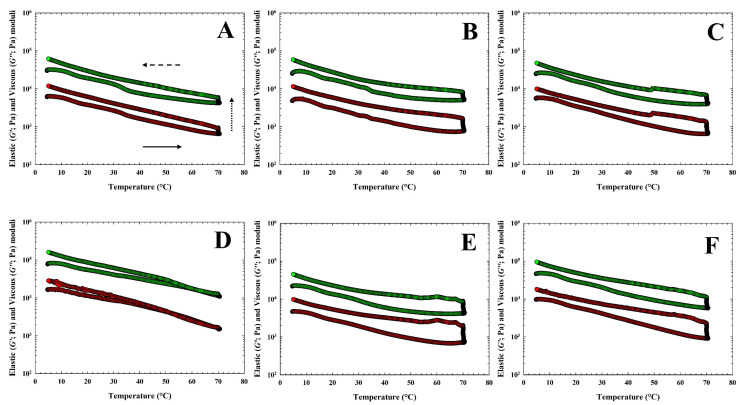
Development of the elastic (G′; green circles) and the viscous (G″; red circles) moduli of chicken liver pâté samples during heating (bottom part of the curve; the direction of the arrow (solid) presents the temperature increase), holding at 70 °C (presented with the dot arrow) and cooling [upper part of the curve; the direction of the arrow (dash) presents the temperature decrease] of the chicken liver pate samples with three different hydrocolloids [(**A**): 0.25% *w*/*w* of κ-carrageenan (ΚΚ) and (**D**): 1.00% *w*/*w* of κ-carrageenan; (**B**): 0.25% *w*/*w* of ι-carrageenan (IK) and (**E**): 1.00% *w*/*w* of ι-carrageenan; (**C**): 0.25% *w*/*w* of furcellaran (FR) and (**F**): 1.00% *w*/*w* of furcellaran].

The obtained values of elastic (G′) and viscous (G″) moduli of CLP samples during heating, holding at 70 °C, and cooling of the CS and for CLP samples with three different hydrocolloids are shown in Figure 4 and Figure 5. In Table 4, the values of G′, G″, and tan δ at temperatures of 70 °C (heating), 20 °C, and 5 °C (cooling) are given. In general, for CLP samples with the application of κ-carrageenan, the values of G′ and G″ had the highest effect with the addition of 0.75% (*w*/*w*) and the lowest effect with the addition of 0.25% (*w*/*w*) (*p* < 0.05). Increasing the amount of κ-carrageenan led to an increase in the values of G′ and G″, which is evident in the increase from a value of 0.75% *w*/*w* at a temperature of 5 °C (*p* < 0.05). The values of G′ and G″ at a temperature of 20 °C appeared to increase with the effect of temperature in CLP samples following the addition of 0.25% *w*/*w* κ-carrageenan. When comparing individual groups of CLP samples, the highest values of G′ and G″ were achieved at 5 °C and 20 °C for products with the addition of 1.00% *w*/*w* furcellaran (*p* < 0.05).

During the cooling stage, the values of G′ and G″ gradually increased (*p* < 0.05). According to Feng et al. [47] this may probably be due to the continued occurrence of cross-linkings between the protein molecules, indicating that the gel structure of the CLP samples was further strengthened during cooling. Additionally, Feng et al. [47] mentioned incorporating different types of hydrocolloids, including carrageenan, as the most effective strategy to improve the quality profiles of emulsified meat products. Moreover, the above-mentioned authors [47] reported that it is important to investigate the effects of non-covalent interactions on the quality of meat products before and after heat treatment. In their previous work [49], they found a positive effect of κ-carrageenan on the textural properties of sausages. As mentioned by Javadi et al. [50], using hydrocolloids is a possibility to modulate both the phase behavior and support the stability of the mass mixture. Non-covalent interactions (e.g., hydrogen bonds, hydrophobic interactions, ionic bonds, and Van der Waals forces) or physical linkage between meat proteins and carrageenan are mainly influenced by the incorporation forms and concentrations of carrageenan. In addition, Cao et al. [49], reported, that intermolecular linkage through hydrogen bonds or ionic interactions between meat proteins and carrageenan could improve the rheological properties of meat batters.

Another indicator describing changes in rheological properties are the tan δ values. In our study, the tan δ values of all samples were lower than 1, indicating that the samples presented a more elastic character. Tan δ values of CLP samples at three different temperatures from the heating-holding-cooling cycle are shown in Table 4. The highest rate of decrease in the tan δ value was found for the sample with 1.00% κ-carrageenan at 70 °C (*p* < 0.05). The resulting structure of the latter CLP sample thus, came close to ideal elastic behavior. Cao et al. [49] reported that a lower value of tan δ at the end of the heat treatment is usually related to a higher elasticity. When evaluating the obtained tan δ values for individual types of hydrocolloid and concentrations, no significant trends could be traced between individual samples (*p* ˃ 0.05). In particular, the tan δ values of the tested CLP ranged from 0.13 ± 0.03 to 0.24 ± 0.01. Le et al. [51] reported that heat treatment is a process of stabilizing gels with a protein-polysaccharide mixture by inducing the formation of additional bonds (e.g., by accumulating proteins on the initial network backbone after heating). In general, the results obtained are in accordance with those previously reported by Delgado et al. [31], Steen et al. [52], Kumar et al. [53], and Polášek et al. [9].

### 3.4. Instrumental Color Analysis

Instrumental color analysis results are shown in Table 5. The CLP samples with hydrocolloids were slightly darker compared to the CS (*p* < 0.05). The L* values of the CLP samples ranged from 53.10 ± 0.25 to 57.45 ± 0.01. Furthermore, the a* values ranged from 11.88 ± 0.03 to 13.70 ± 0.05, and the b* values ranged from 14.69 ± 0.16 to 15.52 ± 0.04. We attribute the color properties and the influence on them to the product’s own raw material composition, the type and proportion of components of animal origin (liver and their higher content of blood dyes), rather than the influence of the hydrocolloid used. On the contrary, the L*, a*, b* values of our model CLP samples were similar to the L*, a*, b* values of the products (Bologna sausage) reported by Pirres et al. [47]. Moreover, the studies of Delgado et al. [31] and Estévez et al. [54] have presented similar results. Overall, it can be concluded that the amount and type of hydrocolloid used did not influence the CLP color properties.

## 4. Conclusions

The effect of κ-, ι-carrageenan and furcellaran (at 0.25, 0.50, 0.75 and 1.00% *w*/*w*) addition on the bchicken liver pâté samples’ physicochemical, textural and rheological properties was evaluated. Increasing concentrations of all tested hydrocolloids led to an increase in the values of hardness, spreadability and the viscoelastic moduli of the chicken liver pâtés. In general, κ-carrageenan was evaluated to be a more effective thickening agent than ι-carrageenan and furcellaran. Furthermore, all chicken liver pâté samples presented a more elastic character (G′ ˃ G″). The values obtained by instrumental analysis of color indicated that all samples (regardless of the applied hydrocolloid type or concentration) could be characterized as chicken liver pâtés of a red color with a weak yellow tint. A practical benefit of the current study could be considered that for consumers who prefer chicken liver pâtés with a softer consistency, a concentration of furcellaran ≤ 0.75% *w*/*w* and ι-carrageenan ≤ 0.25 *w*/*w* could be recommended. On the other hand, for consumers who prefer firmer product consistency, it is possible to apply κ-carrageenan in concentrations ≥0.25% *w*/*w* and ι-carrageenan in concentration ≥0.50% *w*/*w*. On the whole, the application of algal hydrocolloids proved to be an effective way to modify the techno-functional properties of chicken liver pâtés.

The effect of algal hydrocolloids on the techno-functional properties of chicken liver pâté can be considered interesting in terms of further research, considering the limited number of available studies on this issue. Hence, a further follow-up study could be the evaluation of hydrocolloid mixture addition on the techno-functional and organoleptic properties of chicken liver pâté samples during storage.

## Figures and Tables

**Table 1:** Raw material formulation of chicken liver pâté samples.

Raw Materials	Ingredients Composition (% *w*/*w*)				
	CS	CLP_0.25	CLP_0.50	CLP_0.75	CLP_1.00
Chicken liver	31.00	30.75	30.50	30.25	30.00
Chicken thigh filet	25.50	25.50	25.50	25.50	25.50
Chicken skin	24.00	24.00	24.00	24.00	24.00
Chicken broth	17.00	17.00	17.00	17.00	17.00
Hydrocolloid *	none	0.25	0.50	0.75	1.00
Nitrite curing salt **	2.00	2.00	2.00	2.00	2.00
Seasoning mixture	0.50	0.50	0.50	0.50	0.50

* Utilized three types of hydrocolloids: κ-carrageenan (KK), ι-carrageenan (IK) and furcellaran (FR). ** Consisting of sodium chloride (98% *w*/*w*), sodium tripolyphosphate, sucrose, sodium nitrite (a 0.3% *w*/*w* maximum content of NaNO_2_) and dextrose. CS: control sample; CLP_0.25: chicken liver pâté with 0.25% *w*/*w* hydrocolloid addition; CLP_0.50: chicken liver pâté with 0.50% *w*/*w* hydrocolloid addition; CLP_0.75: chicken liver pâté with 0.75% *w*/*w* hydrocolloid addition; CLP_1.00: chicken liver pâté with 1.00% *w*/*w* hydrocolloid addition.

**Table 2:** Values of moisture, fat and protein contents, pH, and water activity (a_w_) of the model chicken liver pâté samples (*n* = 6). *^,^**.

Hydrocolloid Type ***	Concentration (% *w*/*w*)	Moisture Content (% *w*/*w*)	Fat Content (% *w*/*w*)	Protein Content (% *w*/*w*)	pH	a_w_	Emulsion Stability (% rel.)
CS	none	70.68 ± 0.16 ^a,A^	40.17 ± 0.71 ^a,A^	15.06 ± 0.01 ^a,A^	6.93 ± 0.02 ^a,A^	0.994 ± 0.001 ^a,A^	99.96 ± 0.01 ^a,A^
KK	0.25	70.65 ± 0.14 ^a,A^	39.92 ± 0.08 ^a,A^	14.56 ± 0.08 ^a,A^	6.93 ± 0.01 ^a,A^	0.998 ± 0.001 ^a,A^	99.97 ± 0.02 ^a,A^
	0.50	70.32 ± 0.05 ^a,A^	38.28 ± 0.62 ^a,A^	14.52 ± 0.06 ^a,A^	6.91 ± 0.01 ^a,A^	0.996 ± 0.001 ^a,A^	99.32 ± 0.04 ^a,A^
	0.75	69.97 ± 0.12 ^a,A^	40.59 ± 0.41 ^a,A^	14.60 ± 0.08 ^a,A^	6.93 ± 0.01 ^a,A^	0.997 ± 0.001 ^a,A^	99.86 ± 0.02 ^a,A^
	1.00	70.34 ± 0.11 ^a,A^	38.53 ± 0.24 ^a,A^	14.50 ± 0.11 ^a,A^	6.94 ± 0.02 ^a,A^	0.994 ± 0.002 ^a,A^	99.33 ± 0.08 ^a,A^
IK	0.25	70.35 ± 0.09 ^a,A^	39.68 ± 0.25 ^a,A^	14.75 ± 0.01 ^a,A^	6.91 ± 0.01 ^a,A^	0.998 ± 0.001 ^a,A^	99.48 ± 0.07 ^a,A^
	0.50	70.24 ± 0.08 ^a,A^	40.03 ± 0.54 ^a,A^	14.36 ± 0.03 ^a,A^	6.90 ± 0.02 ^a,A^	0.995 ± 0.002 ^a,A^	99.89 ± 0.01 ^a,A^
	0.75	70.14 ± 0.12 ^a,A^	37.35 ± 0.28 ^a,A^	14.25 ± 0.09 ^a,A^	6.89 ± 0.03 ^a,A^	0.998 ± 0.001 ^a,A^	99.89 ± 0.02 ^a,A^
	1.00	69.99 ± 0.08 ^a,A^	39.16 ± 0.75 ^a,A^	14.57 ± 0.05 ^a,A^	6.87 ± 0.02 ^a,A^	0.996 ± 0.002 ^a,A^	99.94 ± 0.01 ^a,A^
FR	0.25	70.46 ± 0.14 ^a,A^	40.84 ± 0.25 ^a,A^	14.36 ± 0.06 ^a,A^	6.94 ± 0.01 ^a,A^	0.995 ± 0.002 ^a,A^	99.81 ± 0.06 ^a,A^
	0.50	70.31 ± 0.11 ^a,A^	40.77 ± 0.45 ^a,A^	14.25 ± 0.03 ^a,A^	6.91 ± 0.02 ^a,A^	0.997 ± 0.001 ^a,A^	99.88 ± 0.02 ^a,A^
	0.75	69.97 ± 0.14 ^a,A^	39.85 ± 0.35 ^a,A^	14.57 ± 0.01 ^a,A^	6.90 ± 0.01 ^a,A^	0.995 ± 0.001 ^a,A^	99.91 ± 0.03 ^a,A^
	1.00	69.76 ± 0.09 ^a,A^	39.98 ± 0.24 ^a,A^	14.26 ± 0.01 ^a,A^	6.91 ± 0.01 ^a,A^	0.995 ± 0.002 ^a,A^	99.66 ± 0.01 ^a,A^

* Values are presented as the mean ± SD. ** Mean values within a column (difference between hydrocolloid type; comparing the same hydrocolloid concentration; the control chicken liver pâté sample was also evaluated) followed by different superscript letters statistically differ (*p* < 0.05). Mean values within a column (difference between hydrocolloid concentration, comparing the same hydrocolloid type; the control chicken liver pâté sample was also evaluated) followed by different uppercase letters differ (*p* < 0.05). *** CS: control sample; KK: κ-carrageenan; IK: ι-carrageenan; FR: furcellaran.

**Table 3:** Values of relative adhesiveness, elasticity, cohesiveness and gumminess of the model chicken liver pâté samples (*n* = 6). *^,^**.

Hydrocolloid Type ***	Concentration	Relative Adhesiveness	Elasticity	Cohesiveness	Gumminess
	(% *w*/*w*)		(s)		(N)
CS	none	3.15 ± 0.11 ^a,A^	11.02 ± 0.49 ^a,A^	0.47 ± 0.03 ^a,A^	4.92 ± 0.26 ^a,A^
KK	0.25	3.52 ± 1.53 ^a,A^	11.60 ± 0.68 ^a,A^	0.43 ± 0.03 ^a,A^	7.02 ± 0.38 ^b,B^
	0.50	4.80 ± 2.52 ^a,A^	10.97 ± 0.50 ^a,A^	0.42 ± 0.09 ^a,A^	7.27 ± 1.41 ^b,B^
	0.75	6.72 ± 2.24 ^b,A^	11.79 ± 1.29 ^a,A^	0.34 ± 0.17 ^b,A^	6.20 ± 0.87 ^b,B^
	1.00	9.24 ± 3.26 ^b,A^	11.65 ± 0.15 ^a,A^	0.37 ± 0.02 ^b,A^	6.64 ± 1.78 ^b,B^
IK	0.25	5.41 ± 0.40 ^b,B^	11.31 ± 0.25 ^a,A^	0.42 ± 0.01 ^a,A^	5.22 ± 0.97 ^a,A^
	0.50	4.74 ± 2.02 ^b,B^	11.88 ± 1.05 ^a,A^	0.35 ± 0.09 ^b,A^	4.61 ± 1.50 ^a,A^
	0.75	8.78 ± 1.61 ^b,C^	11.96 ± 0.88 ^a,A^	0.31 ± 0.10 ^b,A^	4.87 ± 2.93 ^a,A^
	1.00	7.94 ± 2.48 ^b,C^	12.02 ± 0.31 ^b,A^	0.28 ± 0.02 ^c,A^	4.29 ± 1.12 ^a,A^
FR	0.25	5.57 ± 2.81 ^a,B^	11.59 ± 0.78 ^a,A^	0.38 ± 0.08 ^a,A^	5.62 ± 1.93 ^a,A^
	0.50	8.50 ± 1.43 ^c,C^	11.61 ± 0.44 ^a,A^	0.37 ± 0.03 ^b,A^	7.35 ± 0.96 ^b,A^
	0.75	8.75 ± 1.56 ^b,C^	11.31 ± 0.51 ^a,A^	0.42 ± 0.04 ^a,A^	8.79 ± 2.57 ^b,A^
	1.00	6.76 ± 2.20 ^b,C^	10.74 ± 0.19 ^a,A^	0.47 ± 0.03 ^a,A^	11.13 ± 1.41 ^c,A^

* Values are presented as the mean ± SD. ** Mean values within a column (difference between hydrocolloid type; comparing the same hydrocolloid concentration; the control chicken liver pâté sample was also evaluated) followed by different superscript letters statistically differ (*p* < 0.05). Mean values within a column (difference between hydrocolloid concentration; comparing the same hydrocolloid type; the control chicken liver pâté sample was also evaluated) followed by different uppercase letters differ (*p* < 0.05). *** CS: control sample; KK: κ-carrageenan; IK: ι-carrageenan; FR: furcellaran.

**Table 4:** Values of elastic modulus (G′; kPa), viscous modulus (G″; kPa) and tan δ during heating and cooling for the temperatures of 70 °C (after holding) and 20 °C and 5 °C (during cooling) of the model chicken liver pâté samples (*n* = 3). *^,^**.

Hydrocolloid Type ***	Concentration	Temperature	G′	G″	tan δ
	(% *w*/*w*)	(°C)	(kPa)	(kPa)	
CS	none	70	68.03 ± 0.08 ^a,A,^_a_	13.98 ± 0.52 ^a,A,^_a_	0.21 ± 0.01 ^a,A,^_a_
		20	283.15 ± 0.11 ^a,B,^_a_	64.99 ± 0.36 ^a,B,^_a_	0.23 ± 0.02 ^a,A,^_a_
		5	575.62 ± 0.05 ^a,C,^_a_	120.75 ± 0.15 ^a,C,^_a_	0.21 ± 0.02 ^a,A,^_a_
KK	0.25	70	61.78 ± 0.21 ^b,B,^_b_	10.27 ± 0.25 ^b,B,^_b_	0.17 ± 0.01 ^b,a,A^
		20	590.61 ± 0.45 ^b,B,^_b_	116.34 ± 0.22 ^b,B,^_b_	0.21 ± 0.02 ^a,A,^_a_
		5	288.24 ± 0.15 ^b,B,^_b_	61.26 ± 0.34 ^b,B,^_b_	0.21 ± 0.03 ^a,A,^_a_
	0.50	70	79.72 ± 0.52 ^c,C,^_c_	14.77 ± 0.26 ^c,C,^_c_	0.19 ± 0.02 ^b,a,A^
		20	350.35 ± 0.54 ^c,C,^_c_	77.03 ± 0.14 ^c,C,^_c_	0.22 ± 0.01 ^a,A,^_a_
		5	682.85 ± 0.21 ^c,C,^_c_	140.35 ± 0.22 ^c,C,^_c_	0.21 ± 0.01 ^a,A,^_a_
	0.75	70	90.49 ± 0.05 ^d,D,^_d_	18.19 ± 0.33 ^d,D,^_d_	0.20 ± 0.02 ^b,a,A^
		20	422.77 ± 0.08 ^d,D,^_d_	87.59 ± 0.21 ^d,D,^_d_	0.21 ± 0.02 ^a,A,^_a_
		5	798.35 ± 0.11 ^d,D,^_d_	158.45 ± 0.17 ^d,D,^_d_	0.20 ± 0.01 ^a,A,^_a_
	1.00	70	126.56 ± 0.26 ^e,E,^_e_	16.41 ± 0.32 ^e,E,^_e_	0.13 ± 0.03 ^c,a,A^
		20	272.94 ± 0.14 ^e,E,^_e_	62.11 ± 0.21 ^e,E,^_e_	0.23 ± 0.02 ^a,A,^_a_
		5	545.08 ± 0.45 ^e,E,^_e_	113.89 ± 0.08 ^e,E,^_e_	0.21 ± 0.01 ^a,A,^_a_
IK	0.25	70	88.69 ± 0.25 ^b,B,^_b_	19.42 ± 0.09 ^b,B,^_b_	0.22 ± 0.02 ^a,A,^_a_
		20	287.74 ± 0.14 _b,B,b_	63.25 ± 0.21 ^b,B,^_b_	0.22 ± 0.01 ^a,A,^_a_
		5	571.21 ± 0.47 ^b,B,^_b_	114.75 ± 0.23 ^b,B,^_b_	0.20 ± 0.02 ^a,A,^_a_
	0.50	70	83.61 ± 0.36 ^c,C,^_c_	18.47 ± 0.14 ^c,C,^_c_	0.22 ± 0.01 ^a,A,^_a_
		20	263.62 ± 0.52 ^c,C,^_c_	61.48 ± 0.63 ^c,C^_,c_	0.23 ± 0.02 ^a,A,^_a_
		5	519.51 ± 0.22 ^c,C,^_c_	112.2 ± 0.15 ^c,C^_,c_	0.22 ± 0.02 ^a,A,^_a_
	0.75	70	105.84 ± 0.08 ^d,D,^_d_	25.26 ± 0.22 ^d,D,^_d_	0.24 ± 0.01 ^a,A,^_a_
		20	278.75 ± 0.09 ^d,D,^_d_	65.98 ± 0.23 ^d,D,^_d_	0.24 ± 0.01 ^a,A^_,a_
		5	541.34 ± 0.45 ^d,D,^_d_	117.2 ± 0.47 ^d,D,^_d_	0.22 ± 0.01 ^a,A,^_a_
	1.00	70	82.16 ± 0.14 ^e,E,^_e_	19.19 ± 0.38 ^e,E,^_e_	0.23 ± 0.02 ^a,A,^_a_
		20	206.55 ± 0.25 ^e,E,^_e_	47.92 ± 0.21 ^e,E,^_e_	0.23 ± 0.02 ^a,A,^_a_
		5	408.45 ± 0.36 ^e,E,^_e_	88.28 ± 0.12 ^e,E^_,e_	0.22 ± 0.02 ^a,A,^_a_
FR	0.25	70	79.84 ± 0.25 ^b,B,^_b_	17.28 ± 0.14 ^b,B,^_b_	0.22 ± 0.01 ^a,A,^_a_
		20	272.91 ± 0.23 ^b,B,^_b_	62.11 ± 0.09 ^b,B,^_b_	0.23 ± 0.01 ^a,A,^_a_
		5	545.21 ± 0.14 ^b,B,^_b_	113.89 ± 0.57 ^b,B,^_b_	0.21 ± 0.02 ^a,A,^_a_
	0.50	70	79.17 ± 0.13 ^c,C,^_c_	15.57 ± 0.63 ^c,C,^_c_	0.20 ± 0.02 ^a,A,^_a_
		20	328.14 ± 0.33 ^c,C,^_c_	70.59 ± 0.25 ^c,C,^_c_	0.22 ± 0.01 ^a,A,^_a_
		5	663.65 ± 0.41 ^c,C,^_c_	131.55 ± 0.78 ^c,C,^_c_	0.20 ± 0.01 ^a,A,^_a_
	0.75	70	95.96 ± 0.29 ^d,D,^_d_	21.18 ± 0.25 ^d,D,^_d_	0.22 ± 0.01 ^a,A^_,a_
		20	332.54 ± 0.18 ^d,D,^_d_	72.96 ± 0.18 ^d,D,^_d_	0.22 ± 0.02 ^a,A,^_a_
		5	661.43 ± 0.14 ^d,D,^_d_	134.95 ± 0.06 ^d,D,^_d_	0.20 ± 0.01 ^a,A,^_a_
	1.00	70	104.19 ± 0.08 ^e,E,e^	19.87 ± 0.26 ^e,E,^_e_	0.19 ± 0.02 ^a,A,^_a_
		20	474.45 ± 0.25 ^e,E,e^	96.64 ± 0.36 ^e,E,^_e_	0.20 ± 0.01 ^a,A,^_a_
		5	916.92 ± 0.18 ^e,E,e^	176.85 ± 0.41 ^e,E,^_e_	0.19 ± 0.02 ^a,A,^_a_

* Values are presented as the mean ± SD. ** Mean values within a column (difference between hydrocolloid type; comparing the same hydrocolloid concentration; the control chicken liver pâté sample was also evaluated) followed by different superscript letters statistically differ (*p* < 0.05). Mean values within a column (difference between hydrocolloid concentration, comparing the same hydrocolloid type; the control chicken liver pâté sample was also evaluated) followed by different uppercase letters differ (*p* < 0.05). Mean values within a column (difference between hydrocolloid concentration, comparing the same hydrocolloid type and temperature; the control chicken liver pâté sample was also evaluated) followed by different subscript letters differ (*p* < 0.05). Each hydrocolloid type was evaluated individually. *** CS: control sample; KK: κ-carrageenan; IK: ι-carrageenan; FR: furcellaran.

**Table 5:** Values of lightness (L*), chromaticity on a green-to-red axis (a*), chromaticity on a blue-to-yellow axis (b*) of the model chicken liver pâté samples (*n* = 6). *^,^**.

Hydrocolloid Type ***	Concentration	L*	a*	b*
	(% *w*/*w*)			
CS	none	58.44 ± 0.04 ^a,A^	12.47 ± 0.05 ^a,A^	14.69 ± 0.16 ^a,A^
KK	0.25	55.60 ± 0.07 ^b,B^	13.11 ± 0.07 ^b,B^	14.97 ± 0.04 ^b,B^
	0.50	56.97 ± 0.13 ^b,C^	12.07 ± 0.28 ^b,C^	15.27 ± 0.02 ^b,C^
	0.75	57.45 ± 0.01 ^b,D^	11.99 ± 0.04 ^b,D^	15.41 ± 0.05 ^b,D^
	1.00	55.40 ± 0.25 ^b,B^	12.89 ± 0.24 ^b,E^	15.21 ± 0.10 ^b,C^
IK	0.25	57.42 ± 0.02 ^c,B^	11.88 ± 0.03 ^c,B^	15.40 ± 0.05 ^c,B^
	0.50	54.76 ± 0.06 ^c,C^	12.94 ± 0.05 ^c,C^	15.34 ± 0.09 ^b,B^
	0.75	54.41 ± 0.31 ^c,C^	13.18 ± 0.17 ^c,D^	15.12 ± 0.09 ^c,B^
	1.00	53.34 ± 0.23 ^c,D^	13.70 ± 0.05 ^c,E^	15.30 ± 0.09 ^b,C^
FR	0.25	55.58 ± 0.10 ^b,B^	13.51 ± 0.19 ^d,B^	14.99 ± 0.05 ^b,B^
	0.50	57.10 ± 0.27 ^b,C^	12.21 ± 0.13 ^d,C^	15.52 ± 0.04 ^c,C^
	0.75	55.58 ± 0.27 ^d,B^	12.89 ± 0.04 ^d,D^	15.34 ± 0.11 ^c,D^
	1.00	53.10 ± 0.15 ^c,D^	11.88 ± 0.03 ^d,E^	15.40 ± 0.08 ^b,D^

* Values are presented as the mean ± SD. ** Mean values within a column (difference between hydrocolloid type; comparing the same hydrocolloid concentration; the control chicken liver pâté sample was also evaluated) followed by different superscript letters statistically differ (*p* < 0.05). Mean values within a column (difference between hydrocolloid concentration; comparing the same hydrocolloid type; the control chicken liver pâté sample was also evaluated) followed by different uppercase letters differ (*p* < 0.05). *** CS: control sample; KK: κ-carrageenan; IK: ι-carrageenan; FR: furcellaran.

## Data Availability

The data presented in this study are available on request from the corresponding author.

## Supplementary Materials

The following supporting information can be downloaded at: https://www.mdpi.com/article/10.3390/ani14182715/s1, Figure S1: Schematic illustration of the manufacturing protocol used for the chicken liver pâté samples.
