# Supplementary material for: Quality Evaluation of Chicken Liver Pâté Affected by Algal Hydrocolloids Addition: A Textural and Rheological Approach
Source: Animals (Basel). 2024 Sep 19;14(18):2715. doi: 10.3390/ani14182715 (PMC11429152; doi:10.3390/ani14182715)
Supplement: Supplementary file 1 [file animals-14-02715-s001.zip › animals-3181134-supplementary.pdf]

Supplementary Materials

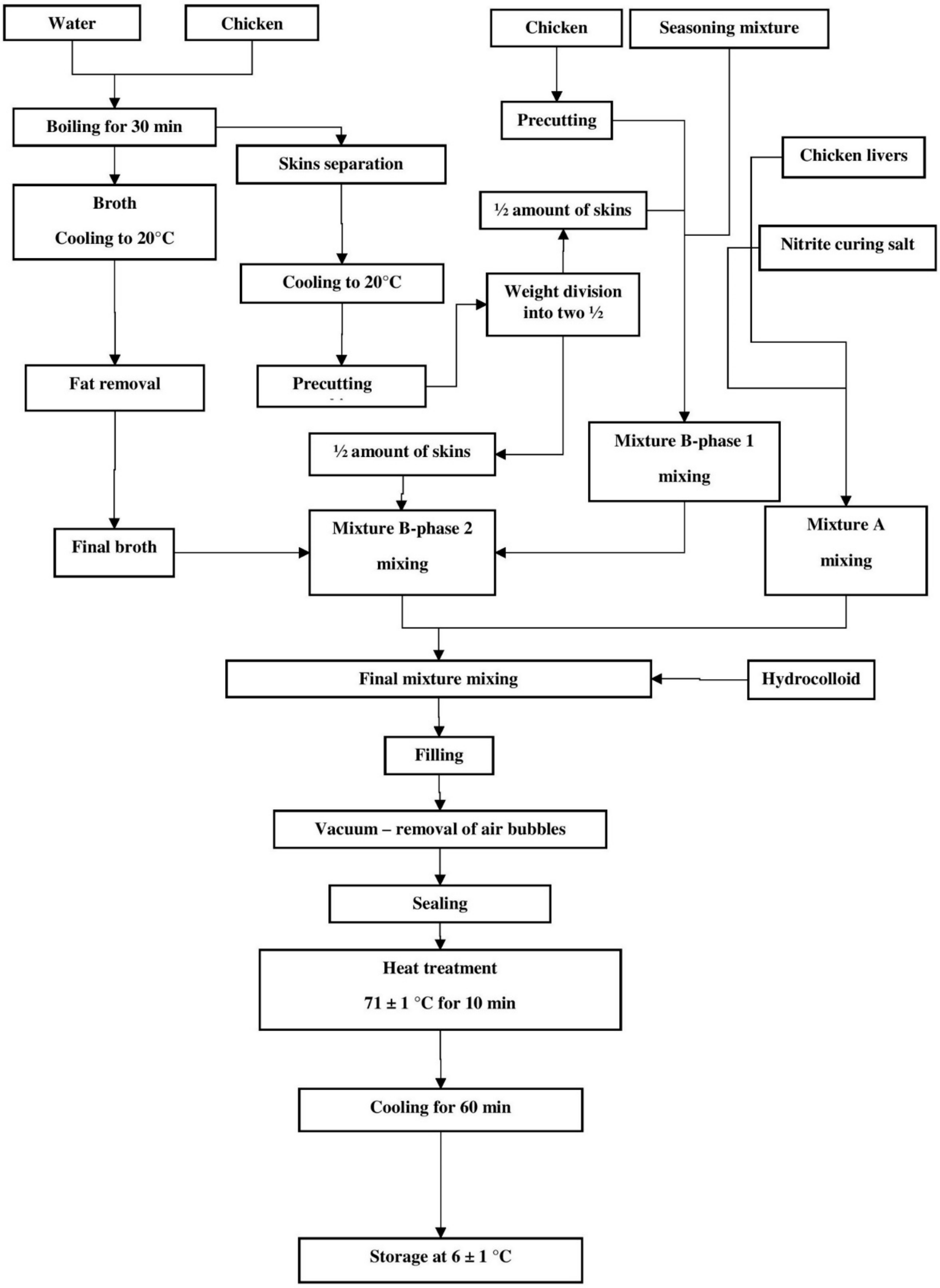

Figure S1. Schematic illustration of the manufacturing protocol used for the chicken liver pâté samples.
